# Saliva microbiome alterations in dental fluorosis population

**DOI:** 10.1080/20002297.2023.2180927

**Published:** 2023-02-20

**Authors:** Shanshan Liu, Qiangsheng Song, Chenchen Zhang, Mengwan Li, Zhenzhen Li, Yudong Liu, Li Xu, Xiaofei Xie, Lili Zhao, Rongxiu Zhang, Qinglong Wang, Guojin Zeng, Yifan Zhang, Kai Zhang

**Affiliations:** a Department of Stomatology, The First Affiliated Hospital of Bengbu Medical College, Bengbu, China; b Anhui Key Laboratory of Infection and Immunity, Bengbu Medical College, Bengbu, China; c Department of Stomatology, Bengbu Medical College, Bengbu, China; d Department of Stomatology, Huaiyuan county people’s Hospital, Bengbu, China; e Department of Histology and Embryology, Bengbu Medical College, Bengbu, China

**Keywords:** Microbiome, dental fluorosis, saliva, population, biomarker

## Abstract

**Background:**

We aimed to explore saliva microbiome alterations in dental fluorosis population.

**Methods:**

The prevalence of dental fluorosis was examined in 957 college students. Dean’s fluorosis index was used to evaluate the dental fluorosis status. Changes in the composition of the salivary microbiome were assessed in a subset of these patients (100 healthy controls, 100 dental fluorosis patients).

**Results:**

Dental fluorosis affected 47% of the student sample, and incidence was unrelated to gender. Compared with healthy controls, the microbiota of patients with dental fluorosis exhibited increased diversity, with increased abundance of *Treponema lecithinolyticum, Vibrio metschnikovii*, *Cupriavidus pauculus*, *Pseudomonas*, *Pseudomonadaceae*, *Pseudomonadales*, and decreased abundance of *Streptococcus mutans*, *Streptococcus sanguinis*, *Gemella*, and *Staphylococcales*. Function analyses showed increases in arginine biosynthesis in patients affected by dental fluorosis, together with reductions in amino sugar and nucleotide sugar metabolism, fructose and mannose metabolism, and starch and sucrose metabolism.

**Conclusions:**

These results suggest that there are striking differences in salivary microbiome between healthy controls and dental fluorosis patients. Dental fluorosis may contribute to periodontitis and systemic lung diseases. There is a need for cohort studies to determine whether altering the salivary microbiota in dental fluorosis patients can alter the development of oral or systemic diseases.

## Background

Fluorine is a non-metallic element that is found at relatively high levels (525 ppm) in the Earth’s crust in the form of fluoride [1]. Although it is a micronutrient required for normal growth, excessive long-term fluoride intake can lead to its deposition in the bones and teeth, resulting in chronic skeletal and dental fluorosis [2,3]. The prevalence of dental fluorosis is rising throughout the world due to higher levels of fluoride exposure, with a prevalence of roughly 89.7% in 12–17-year-olds in Northern Tanzania [4] and 63.7% among 12-year-olds in Quito, Ecuador [5]. The prevalence of dental fluorosis among Mexican 6–12-year-olds and Thai 8–12-year olds is 98% and 53.4%, respectively [6,7], and the average prevalence of dental fluorosis in Jilin, China is 30.5% (range: 8.33% − 64.17%) [8].

Dental fluorosis results from the intake of excessively high fluoride levels through water, soil, brick tea, and coal-based sources via the digestive or respiratory tract, in turn impacting ameloblast development and altering the morphology and mechanical properties of teeth [9]. High fluoride doses can lead to DNA damage, mitochondrial damage, endoplasmic reticulum (ER) stress, and apoptotic death, thereby impairing ameloblast function and disrupting the normal synthesis and secretion of enamel-associated proteins via the impairment of normal ER function [10–13]. When the enamel surface sustained mild or moderate damage, it can change in color, show streaking or the formation of spots, with the appearance of light yellow to brown plaques forming either in limited areas or throughout the enamel surface [14]. These changes can also increase the odds of use-related deterioration and impact chewing and digestive functionality, resulting in tooth sensitivity and other complications as well as presenting difficulties for tooth enamel bonding [15,16]. This can also adversely impact the intellectual development of children, with the most profound effects occurring in more severe cases of dental fluorosis, increasing the risk of developing autism spectrum disorder [17–19].

The microbiota is often studied due to its potential as a disease-related biomarker or target for disease management efforts. A growing number of studies have highlighted the association between fluoride and microbes. For example, the intake of elevated fluoride levels (80 mg/L) can alter both the makeup and diversity of the gut microbiota in common carp, resulting in reduced *Actinobacteria* abundance with a concomitant increase in *Firmicutes* and *Fusobacterium* abundance [20]. While exposure to a 100 mg/L dose of fluoride does not impact the diversity of the rat fecal microbiome, the richness of these intestinal communities initially rises and then falls as the levels of fluoride in the water rise [21]. The oral cavity is a highly diverse microbial habitat in humans and home to a relatively stable microbiome that can be impacted by environmental factors, dietary composition, and overall health status [22]. Few studies to date have examined dental fluorosis-related changes in the oral microbiota [23]. This study sought to evaluate the prevalence of dental fluorosis among college students in Bengbu Medical College, China, with subsequent evaluation of the structural characteristics of the oral microbiome in 100 patients with dental fluorosis and 100 healthy controls.

## Materials and methods

### Study population

The Ethics Committee of XXX (XXX) approved the present study, which was performed at Bengbu medical college in Bengbu, China. The necessary sample size for this study was calculated using the formula N=(Z_α/2_/δ)^2^*P*(1-*P*) in order to ensure that this study was adequately powered to assess dental fluorosis prevalence as per the results of the 4th National Oral Health Survey in China [24]. When the values of α, Z_α/2_, δ, and *P* were set as 0.05, 1.96, 5%, and 13.4%, the minimum sample size required for this study was calculated to be 178. Overall, 957 college students were recruited for this study. Dean’s index was used to record the incidence and severity of dental fluorosis as follows: 0 (normal), 0.5 (questionable), 1 (very mild), 2 (mild), 3 (moderate), and 4 (severe) [24,25]. Of the included study participants, 100 healthy controls and 100 individuals with dental fluorosis were subjected to further studies of the microbiota. For these 200 patients, exclusion criteria included the presence of dental caries, recurrent oral ulcers, periodontitis, other oral diseases, systemic or genetic diseases, or the use of any antibiotics, tobacco, and probiotics within the past six months.

### Saliva collection

The study participants were directed to fast for 2 h and to rinse their mouths to remove any debris before saliva sample collection. In total, 3 mL of saliva was collected per participant, with 1 mL of each sample being used for microbiome analyses. Samples were transported to the laboratory in Bengbu Medical College within four hours of collection and were stored at−80°C prior to subsequent analysis.

### Saliva DNA extraction, 16s rRNA sequencing and analysis

For all analyzed saliva samples, DNA extraction, library preparations, and Illumina NovaSeq sequencing were conducted at Wuhan Metware Biotechnology CO. Ltd. (Wuhan, China). Samples of salivary DNA were extracted via the CTAB/SDS method. Briefly, the frozen samples were thawed on ice and then centrifuged. One milliliter of CTAB and 20 µL of lysozyme were mixed with the saliva sample, followed by complete lysis in a 65°C water bath. After centrifugation at 12,000 rpm for 10 min, 950 µL of the supernatant was mixed with an equal volume of phenol (pH 8.0): chloroform: isoamyl alcohol (V25:24:1). After centrifugation, the supernatant was mixed with an equal volume of chloroform: isoamyl alcohol (V24:1) and re-centrifuged. The supernatant from the centrifugation was then mixed in isopropanol and precipitated at −20°C. The samples were re-centrifuged, the supernatants were discarded, and the precipitates were washed twice with 1 ml of 75% ethanol. After drying at room temperature for 2–3 min, the DNA was dissolved in ddH_2_O, treated with RNase A, and incubated for 15 min at 37°C. 16S rRNA contains nine hypervariable regions (V1-V9). Digvijay Verma et al reported that oral microbiome-based NGS analysis chiefly relies on primers for either the V1–2 or V3–4 regions; these pieces of the 16S rDNA regions are sufficiently capable of providing an entire picture of the bacterial phyla present in a niche [26]. Thus, we chose the V3-V4 hypervariable 16S rRNA gene sequences for analysis in the current study. The 16S rRNA gene V3-V4 region was amplified using the forward (CCTAYGGGRBGCASCAG) and reverse (GGACTACNNGGGTATCTAAT) primers with the barcode [27]. Sequencing libraries were generated using TruSeq® DNA PCR-Free Sample Preparation Kit (Illumina, USA) and sequenced on an Illumina NovaSeq platform. The barcode and primer sequences were removed via Perl v 5. FLASH v 1.2.7 (http://ccb.jhu.edu/software/FLASH/) [28] was used to splice the reads from each sample, with the resultant splicing sequence representing the raw reads. The raw reads were then filtered to obtain high-quality clean reads by fastp (https://github.com/OpenGene/fastp). The chimeric sequences were removed and the effective reads were obtained using VSEARCH (https://github.com/torognes/vsearch/) [29]. The DADA2 was used for resulting in an amplicon sequence variant (ASV) using QIIME 2 with default parameters [30,31]. The taxonomic classification was analyzed against the SILVA v138.1 [32] and National Center for Biotechnology Information (NCBI) databases (https://ftp.ncbi.nih.gov/pub/taxonomy/taxdump.tar.gz). The Chao1 index (http://scikit-bio.org/docs/latest/generated/skbio.diversity.alpha.chao1.html#skbio.diversity.alpha.chao1) for richness and Pielou_e index (http://scikit-bio.org/docs/latest/generated/skbio.diversity.alpha.pielou_e.html#skbio.diversity.alpha.pielou_e) for evenness were estimated using QIIME 2 [31]. ASVs were assigned to the library using classify-sklearn of QIIME2. A random forest model was used to identify the bacteria that were most readily able to discriminate these groups. Receiver operating characteristic curve (ROC) curves were used to assess model accuracy. Changes among the included study groups were assessed via a Non-metric Multi-Dimensional Scaling (NMDS) analysis based on Bray-Curtis distance values. Phylogenetic Investigation of Communities by Reconstruction of Unobserved States 2 (PICRUSt 2) [33] was used to predict the functions of these microbes.

### Statistics

SPSS 20.0 (IBM, NY, USA) was used to examine the relationship between gender and dental fluorosis. Data were compared with a Pearson chi-square test at a theoretical frequency≥5. When the theoretical frequency was<5 but at least 1(≤20% cell), results were analyzed via continuity correction. All other data were analyzed using Fisher’s exact test. *P* < 0.05 was the significance threshold. Metastats [34] was used to assess differences in diversity and taxonomic composition and a corrected *p* < 0.05 was the significance threshold.

## Results

### Dental fluorosis prevalence rates and its relationship to gender

**Figure 1.**
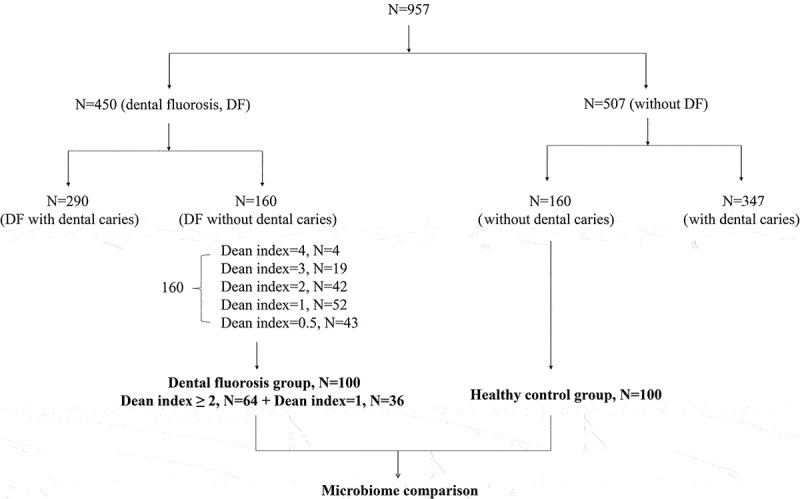
Study design.

The dental fluorosis prevalence among the 957 surveyed college students in Bengbu was 47% (*n* = 450), with respective prevalence rates of 49.8% and 44.7% in males and females. No differences in dental fluorosis rates were observed as a function of gender (*P* = 0.114). Dental fluorosis severity was also found not to be correlated with gender (*P* = 0.976). Of the 450 patients with dental fluorosis, 290 were also affected by dental caries. Of the remaining 160 patients, 43, 52, 42, 19, and 4 exhibited respective Dean index values of 0.5, 1, 2, 3, and 4. A total of 65 patients had a dental fluorosis index greater than or equal to 2, and one of them failed in the sample quality control. The saliva samples of the remaining 64 patients were included in the subsequent microbiome analyses, and 36 patients with a Dean index of 1 were selected at random (61 males and 38 females, respectively). In addition, the healthy control group (HC) comprised of 54 males and 46 females was selected for microbiome analyses. For further details regarding the design of this study, see Figure 1.

### Dental fluorosis patients exhibit substantial variations in the composition of the oral microbiome

**Figure 2.**
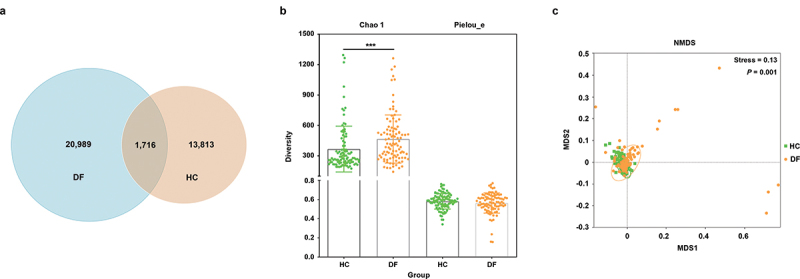
Taxonomic profiles of the oral microbiota in dental fluorosis patients and healthy controls. (a) a Venn diagram corresponding to the ASV in these two groups. (b) α diversity indexes (Chao 1 for richness and Pielou_e for evenness). (c) NMDS analyses of the DF and HC groups based on Bray-Curtis distance values. *** represents *p*-value less than 0.001.

A total of 81,859 average raw reads and 69,173 average effective reads were obtained from the 199 samples. The composition of the oral microbiota in the DF and HC groups was analyzed by collecting salivary samples for the sequencing of the V3-V4 region of the 16S rRNA gene. Overall, 36518 ASVs were identified in the two groups, of which 1,716 were shared between groups while 20,989 and 13,813 were unique to the DF and HC groups, respectively (Figure 2a). The α diversity richness index Chao 1 showed increased diversity in the DF group (*P <* 0.001) while the Pielou_e index showed no significant difference in evenness between the two groups of samples (*P* **=** 0.364) (Figure 2b). β diversity was additionally analyzed based on Bray-Curtis distance values, revealing significant differences in the composition of the oral microbiota in these two groups (*P* = 0.001) (Figure 2c).

### Changes in the salivary microbiome in dental fluorosis population

**Figure 3.**
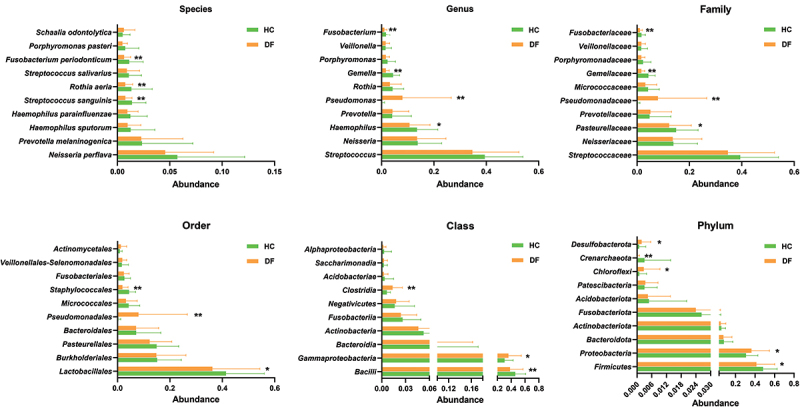
Oral microbiome changes in the 10 most dominant taxa at each level in dental fluorosis population. Metastats was used to assess differences in taxonomic composition. * represents corrected *p*-value less than 0.05. ** represents corrected *p*-value less than 0.01.

To gain further insight into the differential abundance of bacterial taxa between the two groups, the relative bacterial community abundance at each taxonomic level was next assessed, revealing marked differences between the DF and HC groups. When assessing the 10 most dominant species, genera, families, and orders, the DF group exhibited significant increases in *Pseudomonas*, *Pseudomonadaceae*, and *Pseudomonadales* abundance together with reduced *Streptococcus sanguinis*, *Rothia aeria*, *Fusobacterium periodonticum*, *Haemophilus*, *Gemella*, *Fusobacterium*, *Gemellaceae*, *Fusobacteriaceae*, *Pasteurellaceae*, *Lactobacillales*, and *Staphylococcales* abundance relative to the HC group (Figure 3). At the species level, *Streptococcus mutans*, an important cariogenic bacterium, was present at significantly lower abundance in the DF group relative to the HC group (*P* **=** 0.004).

### Dominant members of the salivary microbiome in dental fluorosis population

**Figure 4.**
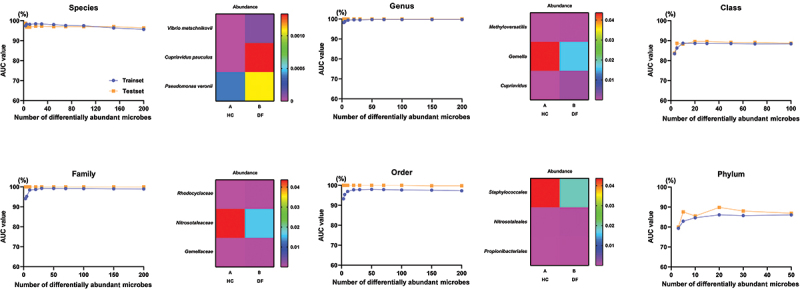
Random forest model analysis. Classification performance of the top three taxa generated by random forest model were assessed by ROC curves. Taxa with AUC values below 0.9 for phylum and class are not displayed.

Next, a random forest model was used to assess specific differences in the composition of the microbiota between these two groups. In total, three species (*Vibrio metschnikovii, Cupriavidus pauculus*, and *Peptostreptococcus stomatis*), three genera (*Methyloversatilis*, *Gemella*, and *Cupriavidus*), three families (*Rhodocyclaceae*, *Propionibacteriaceae*, and *Gemellaceae*), and three orders (*Staphylococcales*, *Propionibacteriales*, and *Vibrionales*) were identified as tentative bacteria capable of differentiating between the DF and HC groups with area under curve (AUC) values of greater than 0.9 (Figure 4).

### Subgroup analysis on gender and severity of dental fluorosis

**Figure 5.**
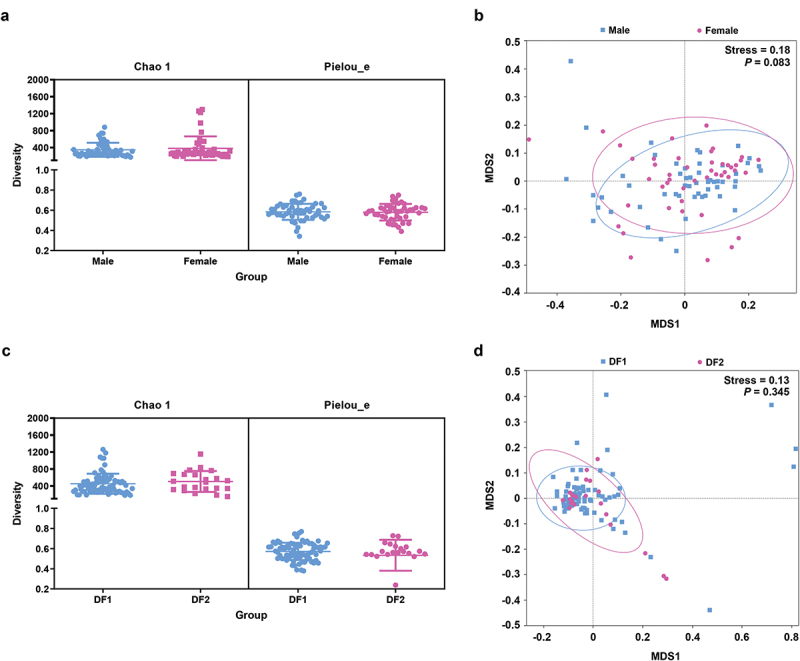
Taxonomic profiles of the oral microbiota in subgroup. (a and c) α diversity indexes (Chao 1 for richness and Pielou_e for evenness). (b and d) NMDS analyses of these groups based on Bray-Curtis distance values.

Dental fluorosis results from the intake of excessively high levels of fluoride in the context of development and tooth growth, and this condition was found to be unrelated to gender as shown above. To assess whether gender was associated with any differences in the makeup of the oral microbiota, the microorganisms present in male and female healthy controls were compared to one another. No differences in any of the analyzed α diversity indexes were evident when comparing males and females (*p* = 0.926 and 0.802 for Chao 1 and Pielou_e, respectively; Figure 5a), nor did β diversity analyses revealed any significant gender-related differences in microbiome composition (*p* = 0.083) (Figure 5b). Thus, gender has no impact on the accuracy of efforts to compare microbes between these populations. We also evaluated whether there was a difference in salivary microbiota between participants with mild dental fluorosis and those with moderate to severe dental fluorosis. The results showed that there was no significant difference in α (Figure 5c) and β diversity (Figure 5d) between the two groups (*p* = 0.234, 0.683, and 0.345 for Chao 1, Pielou_e and NMDS, respectively).

### Functional differences in the salivary microbiome of study groups

**Figure 6.**
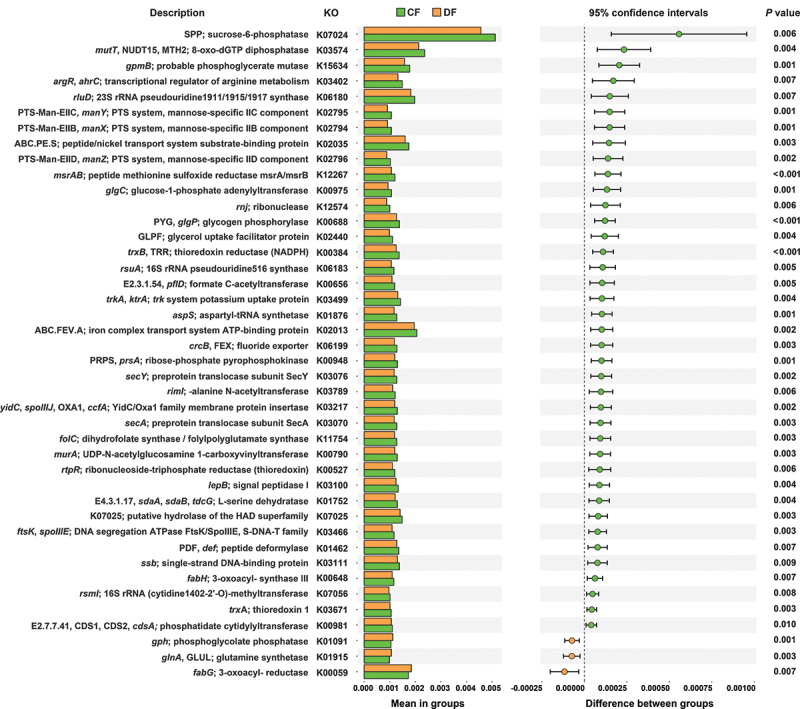
Functional genes identified through PICRUSt 2 analysis. Genes related to taxa with abundance greater than 0.001 and with *p*-values less than 0.01 in either group are shown.

PICRUSt 2 algorithm was used to assess the predicted biological functions of the microbiome in DF and HC group participants, providing a comprehensive overview of differences in salivary ecological characteristics in these groups. The KO entries with *p* value less than 0.01 are illustrated in Figure 6. KO analysis showed a significant difference between the two groups, with most of the functional genes under-represented in the DF group.

## Discussion

Wang et al. previously analyzed saliva samples from 42 adolescents with moderate or severe dental fluorosis, revealing a unique microbial profile in these patients [23]. Owing to limitations in the sample size and disease severity in that study, however, the applicability of these results to other dental fluorosis patients such as those with mild disease remains unclear. Accordingly, in this study a larger cohort of dental fluorosis patients was analyzed, including individuals with mild disease, offering an opportunity to characterize the microbiota associated with this condition. These analyses revealed the oral microflora of dental fluorosis patients to be distinct from those of age-matched control individuals based on 16S rRNA sequencing results. Moreover, a set of bacteria capable of differentiating between dental fluorosis patients and controls was identified.

Alpha diversity is a measure of the richness and evenness of microbial communities, and prior work has revealed that certain diseases are associated with changes in the α diversity of the microbiome, while others are not [35–38]. Zhong et al reported the microbial diversity in fecal samples from rat models of dental fluorosis, revealing an initial increase in such diversity (ACE and Chao indices) with rising NaF levels (25 or 50 mg/L for 12 weeks), whereas this diversity fell when higher levels of NaF were provided in the water given to these rats (100 and 150 mg/L groups) [21]. In the present study, a significant increase in the α diversity in richness of the salivary microbiome was observed in dental fluorosis patients compared with that of control individuals.

Analyses of β diversity revealed significant differences between control individuals and dental fluorosis patients, while there was no significant difference in the saliva microbial diversity between patients with mild and moderate to severe dental fluorosis. The specific differences in bacterial species observed in this study are inconsistent with those in the article published by Wang et al [23]. There may be several reasons for this finding. For one, the age range in the present study was 17–20 years of age, whereas their study included 12–14-year-olds. In addition, their study included 42 participants (9 controls, 33 DF patients), whereas the present study incorporated 199 participants (100 controls, 99 DF patients). There were also differences in the degree of dental fluorosis severity in these studies, with over two-thirds of the participants in the present study exhibiting mild dental fluorosis (mild, Dean index = 1–2, *n* = 76; moderate/severe, Dean index = 3–4, *n* = 23). In contrast, over half of their participants suffered from moderate or severe dental fluorosis (mild, *n* = 14; moderate/severe, *n* = 19). In addition, the difference of the matched databases may also be one of the factors leading to the difference.

The makeup of the oral microbiota is closely associated with both oral and systemic health, and the dysbiosis of this microbial community has been linked to diseases of the oral cavity (dental caries, gingivitis, periodontitis, oral lichen planus, oral cancer), respiratory system (pneumonia, asthma, chronic obstructive pulmonary disease, lung cancer), gastrointestinal system (Crohn’s disease, ulcerative colitis, colonic carcinoma), and other systems [22,39–41]. *Streptococcus mutans* (*S. mutans*) is the bacterium largely responsible for caries formation [42]. Several studies showed that *Streptococcus sanguinis* (*S. sanguinis*) may play an antagonistic role against *S. mutans* colonization and is usually associated with tooth surfaces free of caries [43–45]. However, recently, Al Eraky DM et al reported that *S. mutans* was insignificant contributor to caries among Saudi patients, with caries mostly caused by *S. sanguinis* [46]. Here, significant decreases in the abundance of these species were observed in the DF group, consistent with a lower risk of future caries development. Hu et al. found that tooth biofilm formation in individuals with moderate to severe dental fluorosis (Dean index = 3–4) was associated with differences in *S. mutans* adherence relative to the ability of these microbes to bind the surface of sound enamel [47]. This may be related to the high roughness caused by enamel defects on the surface of dental fluorosis enamel, which provides a better attachment interface for *S. mutans*. This finding may be related to the increased roughness of the enamel in dental fluorosis patients, offering a better interface to which *S. mutans* can attach.

Vandana et al reported that there is a strong association of occurrence of periodontal disease and dental fluorosis [48]. The more serious dental fluorosis is, the rougher the tooth surface is, which makes periodontal bacteria easier to colonize on the tooth surface and difficult for scaling and root planning and may promote the occurrence of periodontal disease [49]. *Treponema lecithinolyticum* is a dominant specie related to the incidence of periodontitis [50]; our study also showed a relative high abundance of *Treponema lecithinolyticum* in the DF group compared with the HC group, suggesting that there may be a potentially high risk of periodontitis in the dental fluorosis population. *Vibrio metschnikovii* (*V. metschnikovii*) and *Cupriavidus pauculus* (*C*. *pauculus*) were dominant species that were more abundant in DF patients and able to discriminate between these patients and those in the HC group. *V. metschnikovii* is an opportunistic pathogen that rarely causes disease onset in humans, whereas *C*. *pauculus* is a non-fermentative, aerobic bacillus that is relatively rare, but both of these species have been reported in lung isolates from humans suffering from severe lung infections [51–54]. These data may suggest that dental fluorosis patients with impaired immune functionality may be at a higher risk of developing opportunistic lung infections. The abundance of *Gemella*, which is a species of Gram-positive bacteria rarely associated with the development of infective endocarditis [55], was reduced in dental fluorosis patients.

Many studies have explored the association between gender and salivary microbiome, and reported that there was gender-specific difference in the salivary microbiome [56,57]. Therefore, in order to determine whether the results obtained in this study are affected by dental fluorosis or gender, we also analyzed the relationship between gender and dental fluorosis, as well as the relationship between gender and the salivary microbiome. No significant gender-related differences in dental fluorosis prevalence were observed, nor were any gender-associated differences in the composition of the oral microbiota in healthy control participants observed, suggesting that gender has no impact on the accuracy of these study results, and that the differential microorganisms identified are indeed closely related to dental fluorosis rather than interfered by gender.

PCRUSt 2 analysis was conducted to predict the mechanism of how fluorosis changes gut or oral bacteria. Strikingly, more than 90% of the functional genes were under-represented in the DF group. Among these under-represented functional genes, K07024 (SPP, sucrose-6-phosphatase), K00688 (*glgP*, glycogen phosphorylase), K00975 (*glgC*, glucose-1-phosphate adenylyltransferase) are involved in starch and sucrose metabolism. K02794 (*manX*, mannose PTS system EIIAB component), K02795 (*manY*, mannose PTS system EIIC component), and K02796 (*manZ*, mannose PTS system EIID component) are involved in fructose and mannose metabolism and amino sugar and nucleotide sugar metabolism. These pathways are associated with active carbohydrate metabolism in the context of cariogenesis [58–60]. Arginine deiminase system (ADS)-positive bacteria can contribute to the alkalization of the oral microenvironment via arginine and proline metabolism, and can inhibit the growth of acidic cariogenic bacterial species such as *S. mutans* [61]. Moreover, arginine is a promising agent for caries management [62]. Arginine biosynthesis is essentially negatively regulated by ArgR/AhrC family proteins. These repressors inhibit arginine synthesis by inhibiting the expression of arginine biosynthetic genes at the transcriptional level [63,64]. Jing et al reported that AhrC is also an important transcription factor that negatively regulates arginine biosynthesis gene expression and bioﬁlm formation in *S. mutans* [65]. In this study, we found that *argR* and *ahrC* (K03402; *argR*, *ahrC*; transcriptional regulator of arginine metabolism) were under-represented in DF group, which suggests that arginine biosynthesis in DF group may be higher than that in HC group, thus correspond to a lower risk of caries in DF patients.

There are some limitations to this study. For one, only college students were recruited, potentially limiting the accuracy and generalizability of the prevalence of dental fluorosis. Thus, the sample size was expanded from 178 to 957 to decrease the experimental error. Additional long-term cohort studies will be essential to explore the potential mechanisms and disease incidence rates in patients with dental fluorosis to more fully understand the pathogenic functions of the oral microbiota and the dysbiosis. A negative relationship between dental fluorosis incidence and the abundance of cariogenic bacteria was observed. Certain opportunistic pathogens associated with lung infections were also observed at high abundance levels in patients with dental fluorosis. Additional studies of disease-related changes in patients with dental fluorosis are warranted to more fully understand the relationship between this condition and other pathogenic outcomes.

## Conclusion

In summary, the present study surveyed structural changes in the composition of different oral microbial communities in dental fluorosis patients and healthy controls. This approach revealed greater microbial diversity in the microbiota of dental fluorosis patients relative to control individuals. Future studies on the oral or systemic disease changes of these dental fluorosis populations deserve further tracking and analysis.

## Data Availability

The data could be obtained upon request to the corresponding author. In addition, microbiome data generated for the 99 dental fluorosis population is available at the National Center for Biotechnology Information (NCBI) under BioProject of PRJNA865814. The Microbiome data generated for the 100 healthy control population is available under BioProject of PRJNA822496.
